# Supplementary material for: Bulk tungsten-substituted vanadium oxide for low-temperature NOx removal in the presence of water
Source: Nat Commun. 2021 Jan 25;12:557. doi: 10.1038/s41467-020-20867-w (PMC7835234; doi:10.1038/s41467-020-20867-w)
Supplement: Supplementary file 1 — Supplementary Information [file 41467_2020_20867_MOESM1_ESM.pdf]

Supplementary Information for

Bulk tungsten-substituted vanadium oxide for low-temperature NOx removal in the presence of water

Yusuke Inomata,[1] Hiroe Kubota,[2] Shinichi Hata,[3] Eiji Kiyonaga,[4] Keiichiro Morita,[4] Kazuhiro Yoshida,[4] Norihito Sakaguchi,[5] Takashi Toyao,[2] Ken-ichi Shimizu,[2] Satoshi Ishikawa,[6] Wataru Ueda,[6] Masatake Haruta,[1] and Toru Murayama[1,7,*]

[1]Research Center for Gold Chemistry, Graduate School of Urban Environmental Sciences, Tokyo Metropolitan University
Hachioji, Tokyo 192-0397, Japan
[2]Institute for Catalysis, Hokkaido University, Sapporo, Hokkaido 001-0021, Japan
[3]Department of Applied Chemistry, Faculty of Engineering, Sanyo-Onoda City University, Sanyo-Onoda, Yamaguchi 756-0884, Japan
[4]Energia Economic and Technical Research Institute, the Chugoku Electric Power Company, Incorporated, Higashi-Hiroshima, Hiroshima 739-0046, Japan
[5]Laboratory of Integrated Function Materials, Center for Advanced Research of Energy and Materials, Faculty of Engineering, Hokkaido University, Sapporo, Hokkaido 060-8628, Japan
[6]Department of Material and Life Chemistry, Faculty of Engineering, Kanagawa University, Yokohama, Kanagawa 221-8686, Japan
[7]Yantai Key Laboratory of Gold Catalysis and Engineering, Shandong Applied Research Center of Gold Nanotechnology (Au-SDARC), School of Chemistry & Chemical Engineering, Yantai University, Yantai 264005, China

*Corresponding author, E-mail: murayama@tmu.ac.jp

Table of contents

Supplementary methods p.S3

Supplementary data

Supplementary Table 1 p.S4
Supplementary Figure 1 p.S5
Supplementary Figure 2 p.S6
Supplementary Table 2 p.S7
Supplementary Figure 3 p.S8
Supplementary Figure 4 p.S9
Supplementary Figure 5 p.S10
Supplementary Figure 6 p.S11
Supplementary Figure 7 p.S12
Supplementary Figure 8 p.S13
Supplementary Table 3 p.S14
Supplementary Figure 9 p.S15
Supplementary Table 4 p.S16
Supplementary Figure 10 p.S17
Supplementary Figure 11 p.S18
Supplementary Figure 12 p.S19
Supplementary Figure 13 p.S20
Supplementary Figure 14 p.S21
Supplementary Figure 15 p.S22
Supplementary Figure 16 p.S23
Supplementary Figure 17 p.S24

Supplementary methods

Catalyst characterization

Thermo gravimetry-differential thermal analysis (TG-DTA) curves were obtained by using HITACHI TG/DTA7200. The specific surface areas of the catalysts were calculated by N_2 adsorption measurements and the Brunauer-Emmett-Teller (BET) equation using MicrotracBEL, BELSORP-max. For N_2 adsorption measurements, 100 mg of the catalyst was put into a quartz sample tube. Infrared (IR) spectra were measured by JES-RE3X (JASCO) equipped with a triglycine sulfate (TGS) detector using the KBr pellet method.

Temperature-programmed reaction measurement

Temperature-programmed reaction (TPR) spectra were obtained by using MicrotracBEL BELCAT. A mass spectrometer (BELMass, MicrotracBEL Corp.) was used for analysis of N_2 gas. Samples (40 mg) were placed in a quartz tube. The samples were heated (20ºC / min) under a gas mixture (500 ppm NH_3 + 500 ppm NO / He) flow.

Supplementary data

Supplementary Table 1. The amounts of reagents for synthesis of W-substituted vanadium oxide (XW-V).

X /	Ammonia metavanadate		Ammonium metatangstate	
mol%	/ g	/ mmol	/ g	/ mmol
0	5.13	43.9	0.00	0.00
1	5.08	43.4	0.11	0.44
3.5	4.95	42.3	0.39	1.53
7	4.87	41.7	0.56	2.19
10	4.77	40.8	0.78	3.07
15	4.36	37.3	1.68	6.58
40	3.08	26.3	4.48	17.5

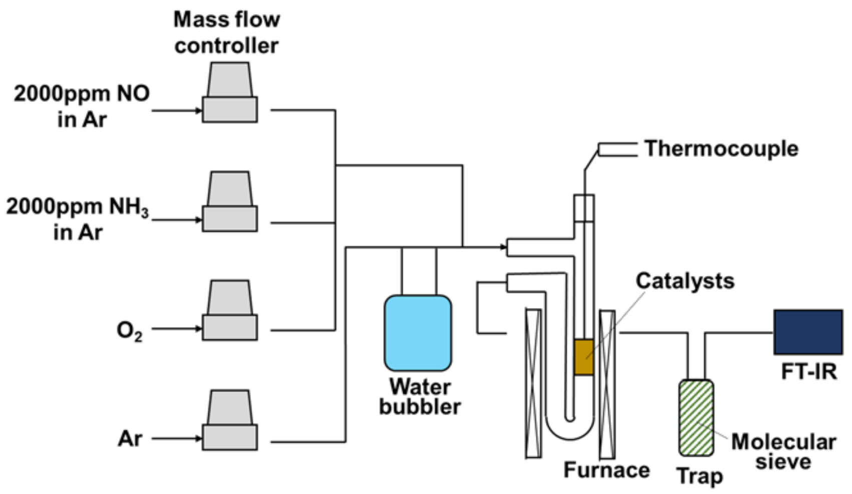

Supplementary Figure 1. **Schematic illustration of a fixed-bed flow reactor system.**

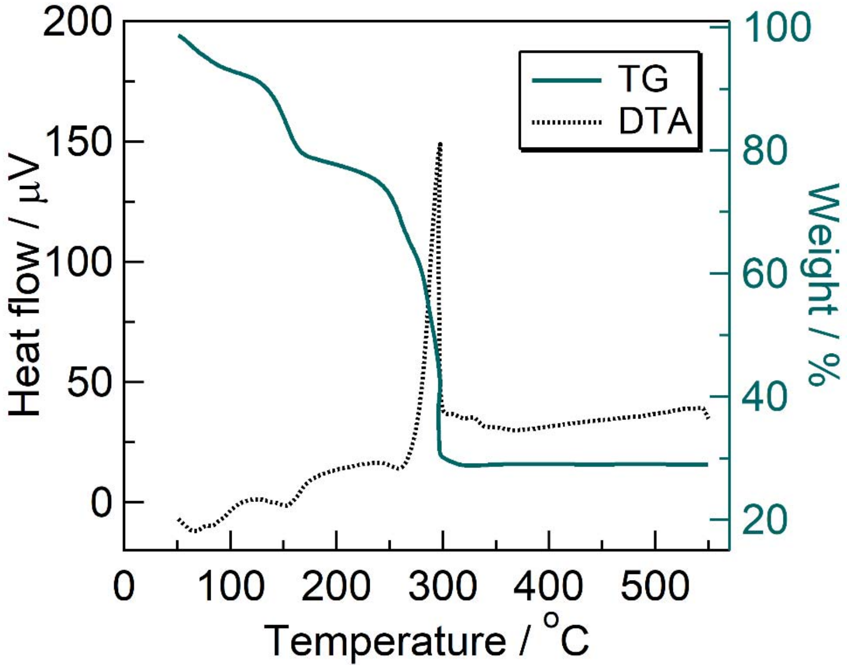

Supplementary Figure 2. TG-DTA curves of the precursor of 3.5W-V.

Supplementary Table 2. Representative experimental parameters of W-substituted vanadium oxide catalysts.

**Catalysts**	**Specific surface area (BET) / $m^2\ g^{-1}$**	**Pore volume / $cm^3 g^{-1}$**	**Averaged pore diameter / nm**
0W-V	41	0.46	16
1W-V	32	0.17	21
3.5W-V	38	0.27	25
7W-V	40	0.22	22
10W-V	36	0.22	24
15W-V	41	0.24	23
40W-V	36	0.14	16
V-W / TiO_2	60	0.41	27

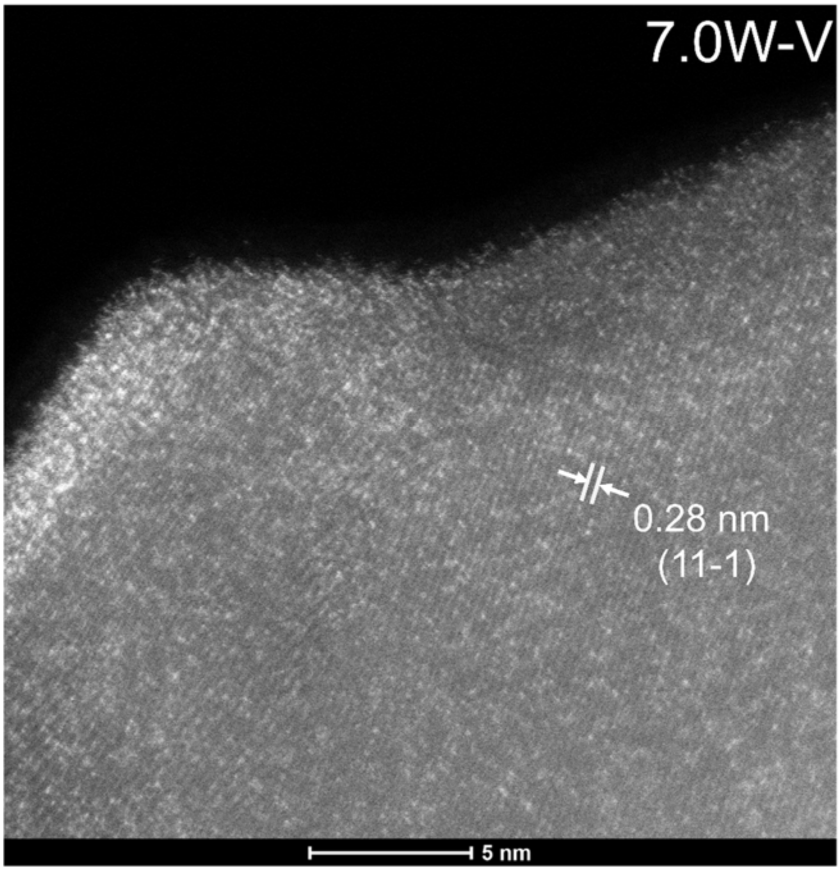

Supplementary Figure 3. Direct observation of tungsten substitution. HAADF-STEM image of 7 mol% W-substituted V_2O_5 (7W-V).

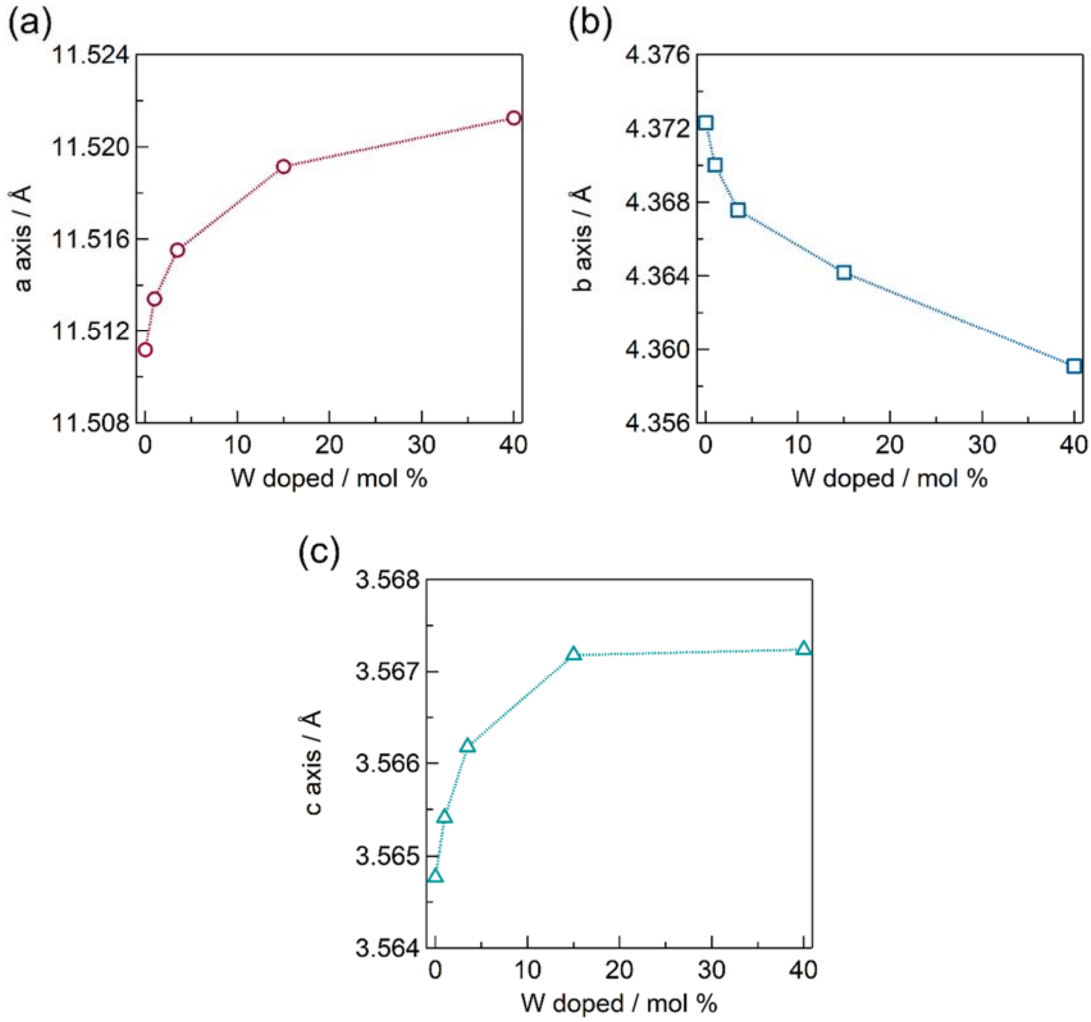

Supplementary Figure 4. **Effect of tungsten substitution on lattice parameters of vanadium oxide.** Lattice parameters for (a) *a*- axis, (b) *b*- axis and (c) *c*-axis as a function of the amount of tungsten doped calculated by Rietveld analysis.

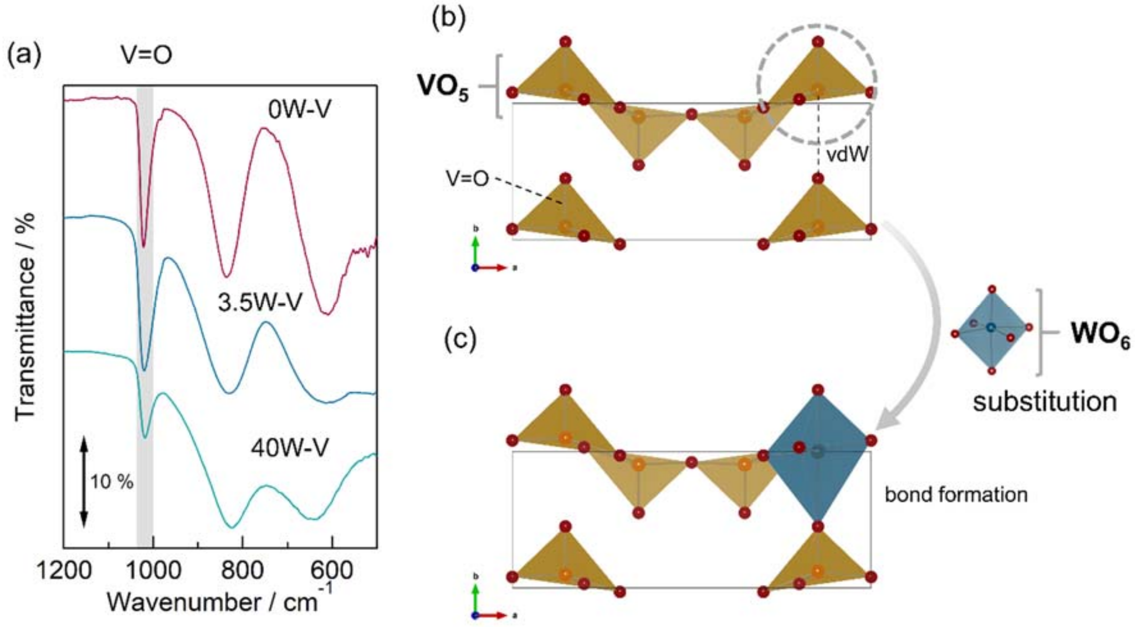

Supplementary Figure 5. **Effect of tungsten substitution on chemical bonds in vanadium oxide.** (a) IR spectra of 0W-V, 3.5W-V and 40W-V. (b) Crystal structure of vanadium oxide and (c) possible structure of W-substituted vanadium oxide. VdW: van der Waals force.

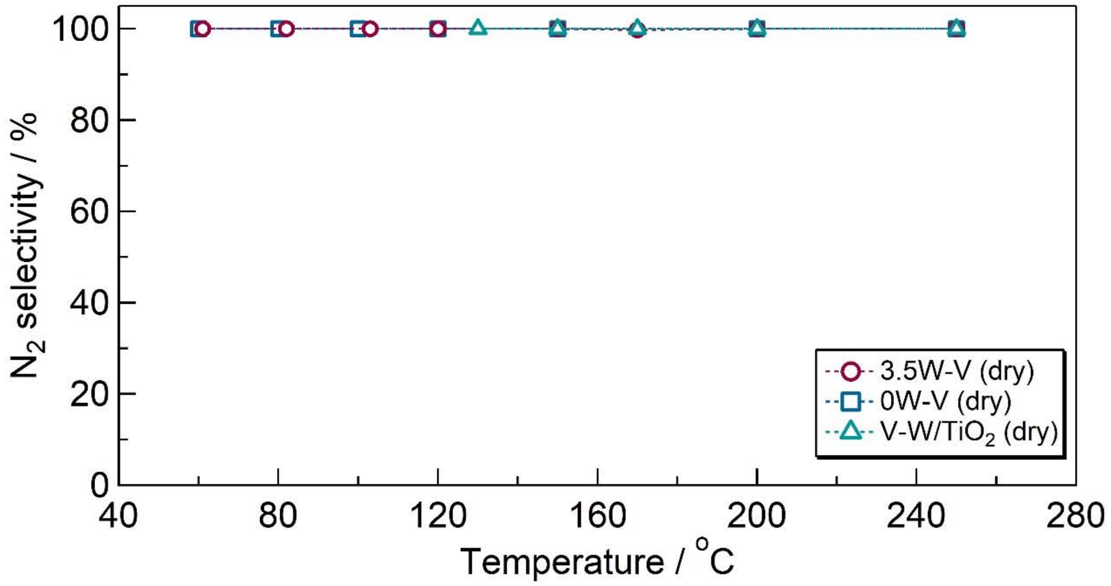

Supplementary Figure 6. Selectivity of catalysts. N_2 selectivity of 3.5W-V (W-substituted vanadium oxide), 0W-V (vanadium oxide only) and V-W/TiO_2 (model of a conventional catalyst) under a dry condition as a function of reaction temperature. Reaction conditions: amount of the catalyst, 0.375 g; reaction gas mixture, 250 ppm NO, 250 ppm NH_3 and 4% O_2 in Ar; flow rate, 250 mLmin^{-1}; space velocity, 40000 mLh^{-1}g$_{cat}^{-1}$.

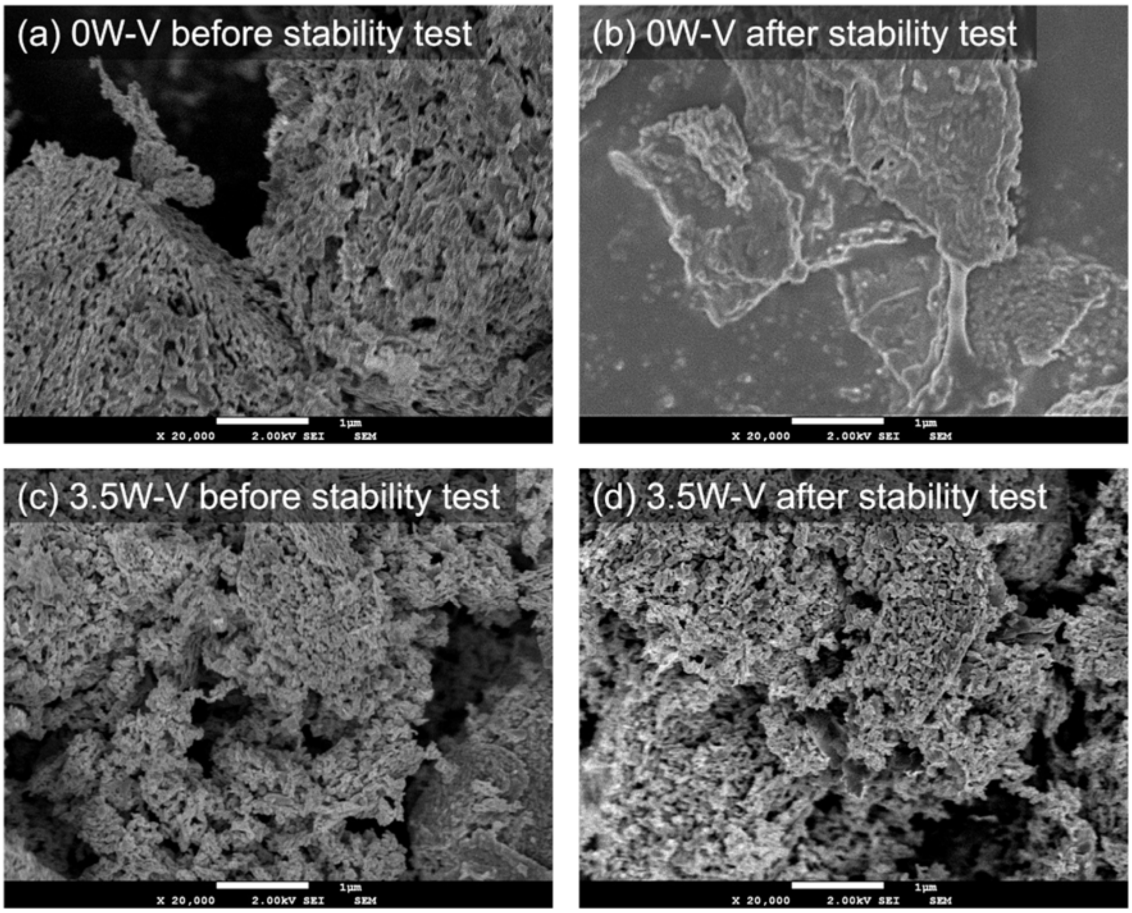

Supplementary Figure 7. Stability of W-substituted vanadium oxide catalysts. SEM images of (a, b) 0W-V and (c, d) 3.5W-V before and after stability test. No morphological changes were observed for 3.5W-V. Although 0W-V had a pore-like structure before stability test, the morphology became smooth after the test.

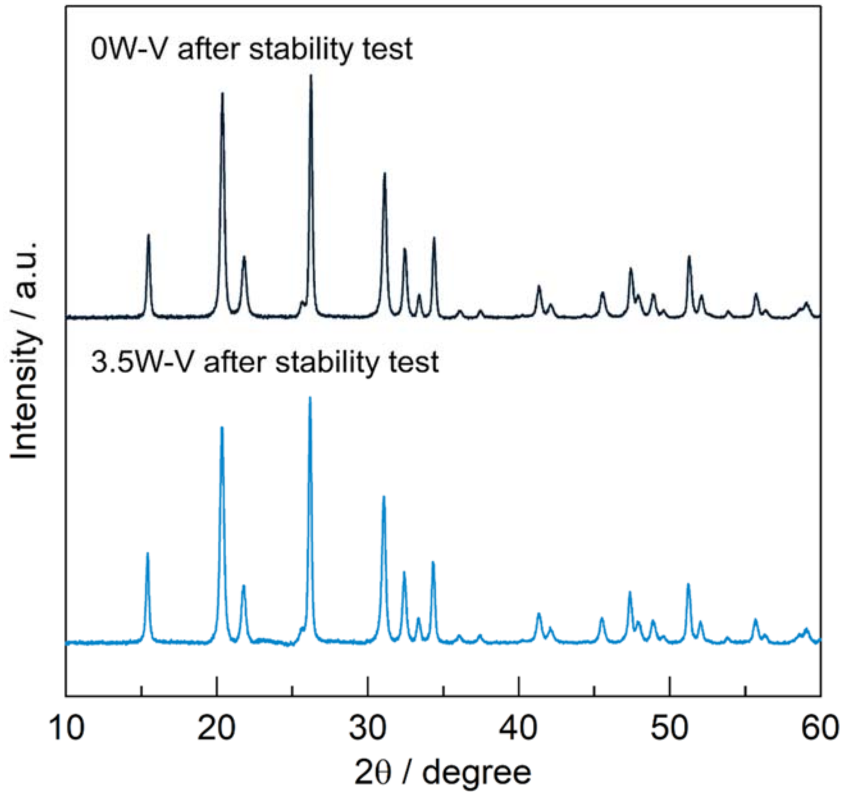

Supplementary Figure 8. Stability of W-substituted vanadium oxide catalysts. XRD patterns of 0W-V and 3.5W-V after stability test. All of the peaks were assigned to V_2O_5 (00-009-0387). XRD patterns before stability test corresponded to that in figure 2a. Crystal structures did not change before and after stability test.

Supplementary Table 3. Reaction rates and apparent activation energy (*E_a*) values of the catalysts.

Catalyst	Reaction rate (150ºC) 10^{-9} / mol_{NO} s^{-1} m^{-2}		Apparent activation energy (E_a) / kJ mol^{-1}	
	Dry	Wet	Dry	Wet
0W-V	3.2	1.4	39	22
3.5W-V	5.2	4.2	36	40
V-W/TiO_2	0.2	0.2	43	49

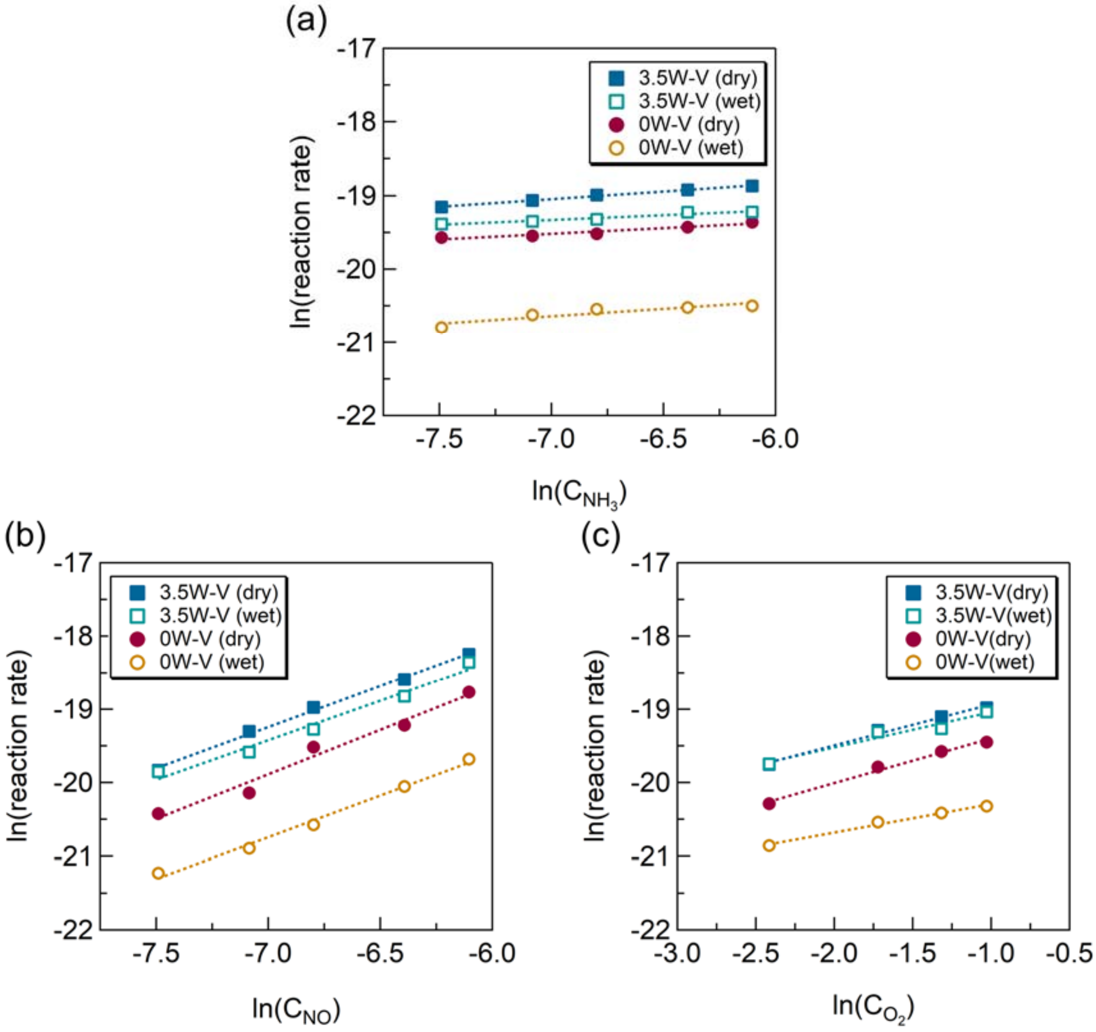

Supplementary Figure 9. Kinetic measurements for NH_3-SCR. Reaction orders (150°C) of 0W-V and 3.5W-V with respect to (a) NH_3 (b) NO and (c) O_2 under a dry condition and wet (10 vol.% water) condition. Ranges of gas concentrations: NH_3, 125-500 ppm; NO, 125-500 ppm; O_2, 2-8%. Reaction rate was measured at an NO conversion below 20% by adjusting the amount of catalysts and the flow rate of reaction gas.

Supplementary Table 4. Reaction orders of vanadium oxide catalysts for NH_3-SCR (150°C).

**Catalysts**	**NH_3**		**NO**		**O_2**	
	Dry	Wet	Dry	Wet	Dry	Wet
0W-V	0.3	0.2	1.0	1.1	0.3	0.4
3.5W-V	0.3	0.2	0.9	1.1	0.3	0.3

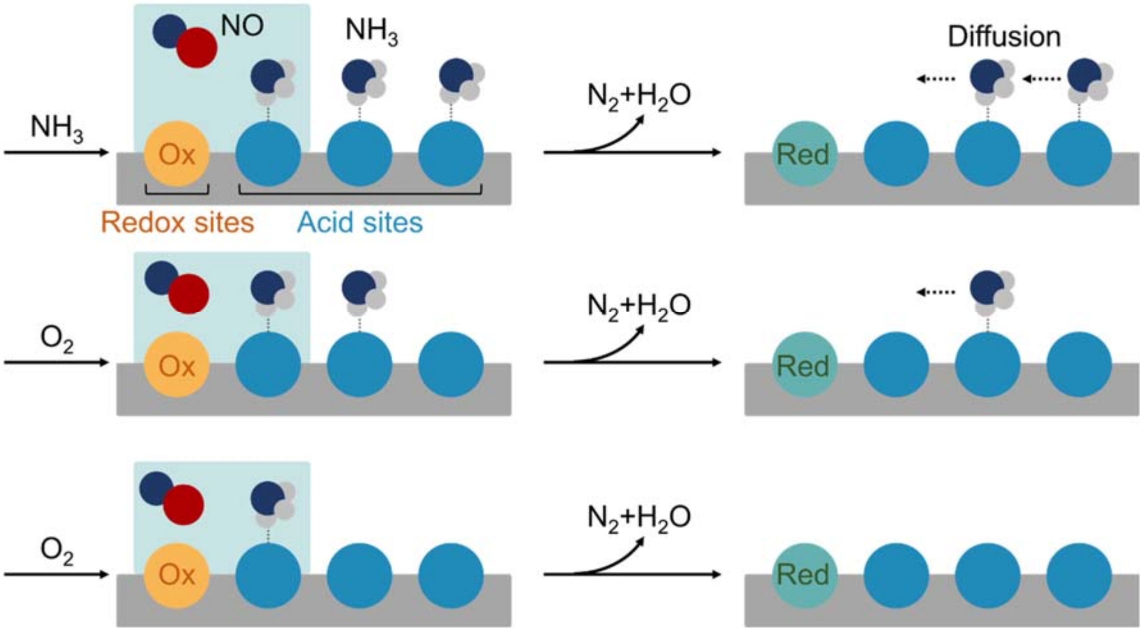

Supplementary Figure 10. *Operando* IR measurement under NO+O_2 exposure. Schematic illustration of *Operando* IR measurement during NH_3 adsorption followed by NO+O_2 exposure.

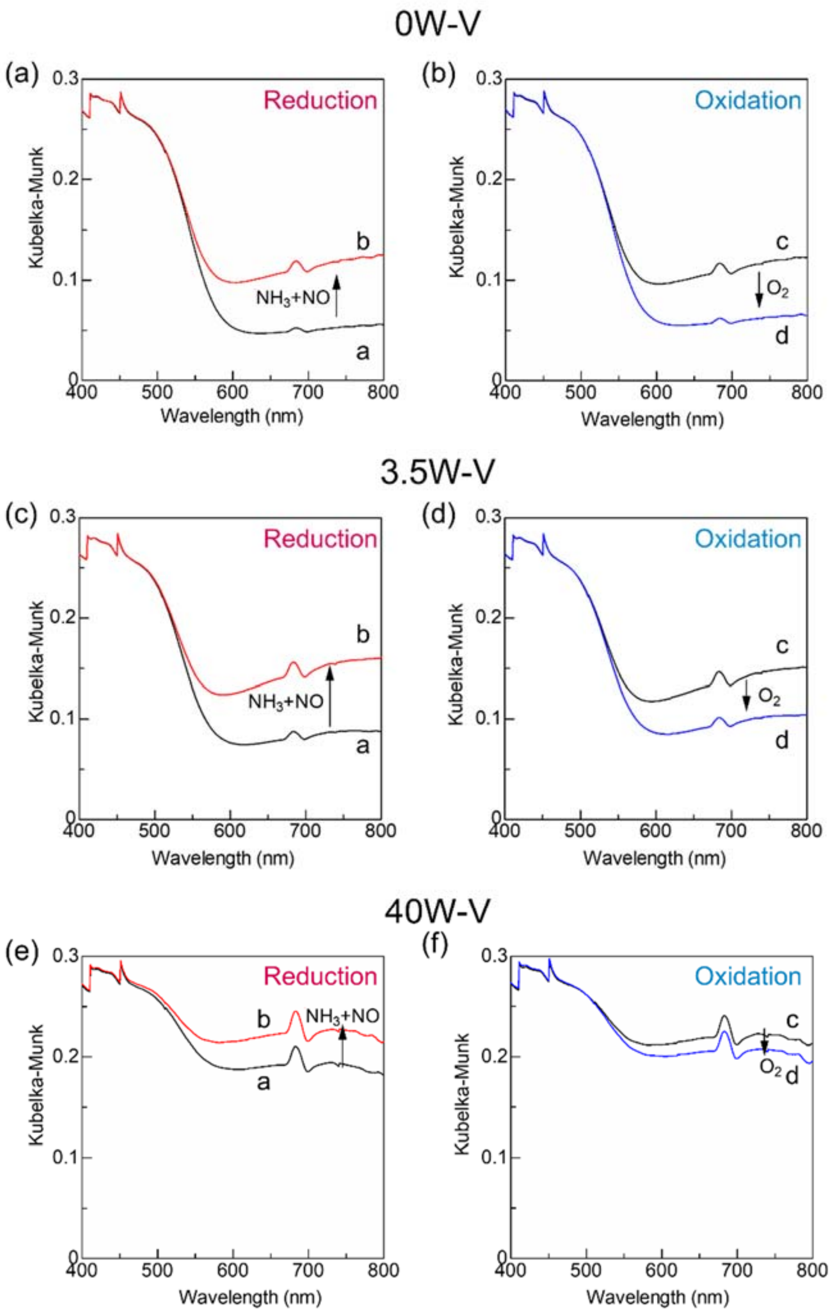

Supplementary Figure 11. ***Operando*** **UV-Vis observation of W-substituted vanadium oxide.** *Operando* UV-Vis spectra for (a, c, e) reduction (500 ppm NO + 500 ppm NH_3) and (b, d, f) oxidation (8% O_2) half cycles at 150ºC under a dry atmosphere for 0W-V (without tungsten), 3.5W-V (with tungsten) and 40W-V (excess tungsten). The characters a-d correspond to the points at which the UV-Vis spectra were observed (see Supplementary Figure 8).

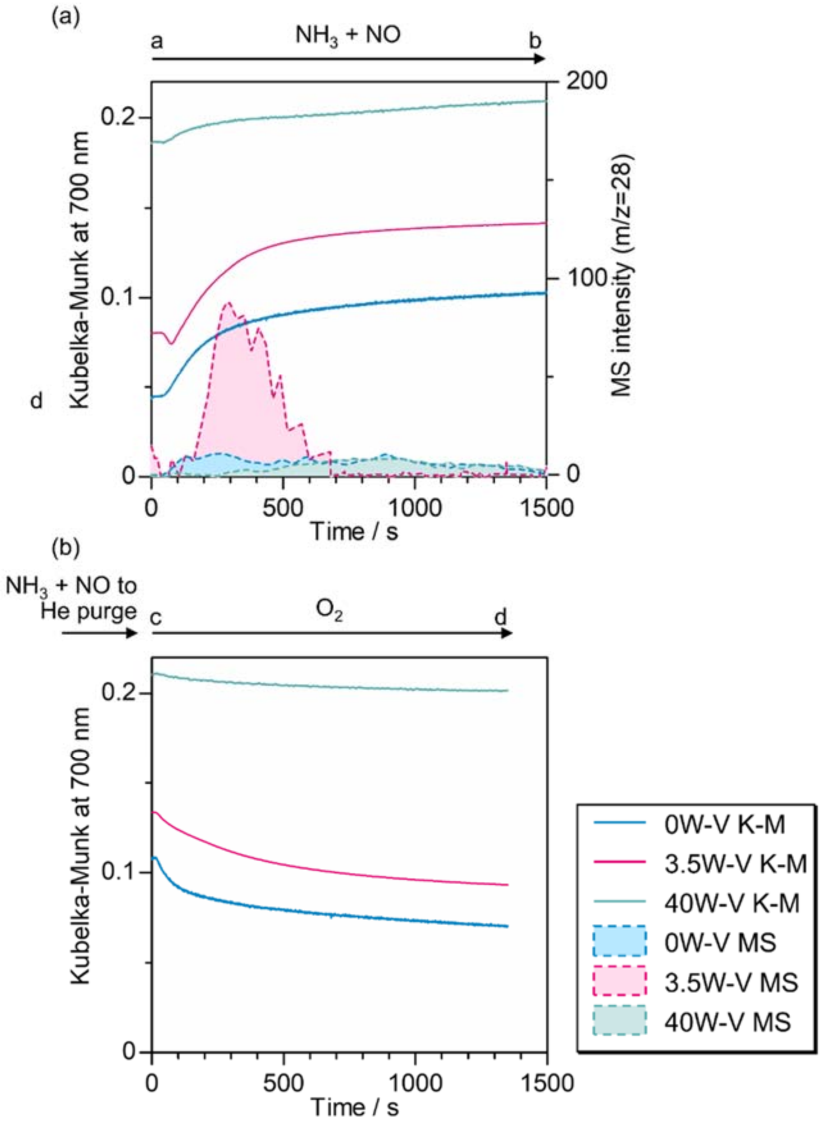

Supplementary Figure 12. Redox properties of W-substituted vanadium oxide. (a) Change in the Kubelka-Munk unit at λ=700nm and MS intensity of N_2 as a function of time during NO (500ppm) +NH_3 (500ppm) flowing at 150ºC under a dry condition for 1500 s. (b) Change in ΔKM_{700} as a function of time during O_2 (8%) flowing at 150ºC under a dry atmosphere for 1350 s. The characters a-d correspond to the points at which the UV-Vis spectra (Supplementary Figure 7) were observed.

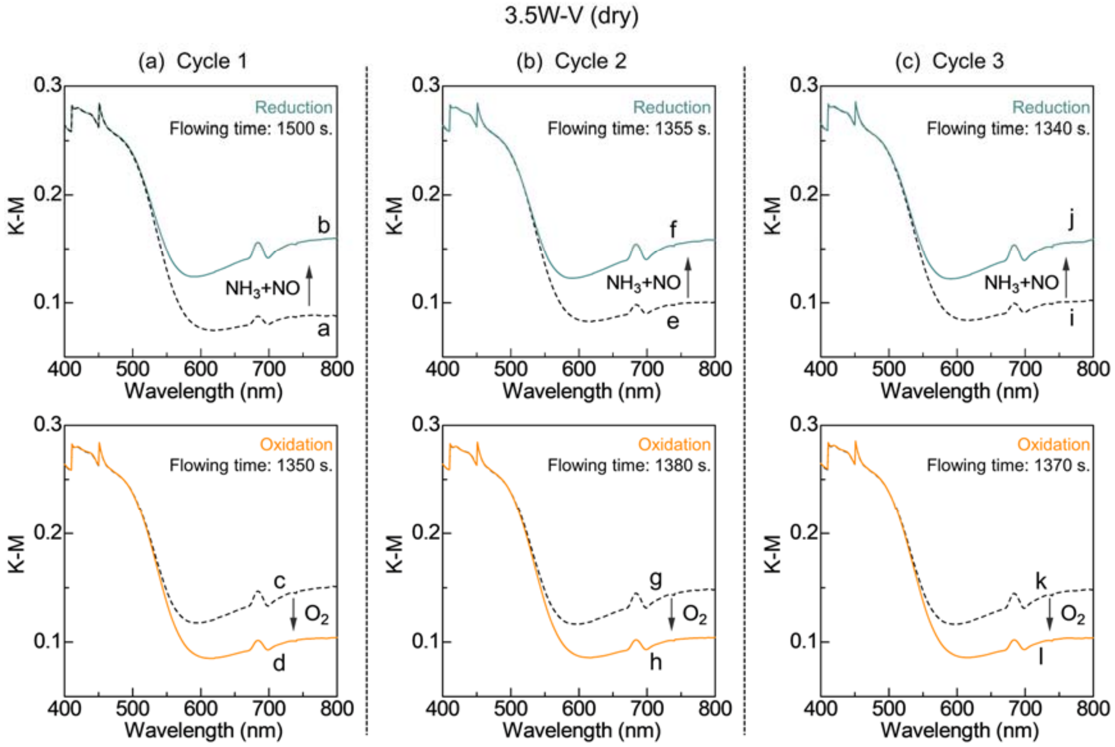

Supplementary Figure 13. ***Operando*** **UV-Vis observation of 3.5W-V for three cycles.** *Operando* UV-Vis spectra of 3.5W-V for reduction (500 ppm NO + 500 ppm NH_3) and oxidation (8% O_2) half cycles at 150ºC under a dry atmosphere for the (a) first cycle, (b) second cycle and (c) third cycle. The characters a-l correspond to the points at which the UV-Vis spectra were observed (see figure 9d in the manuscript).

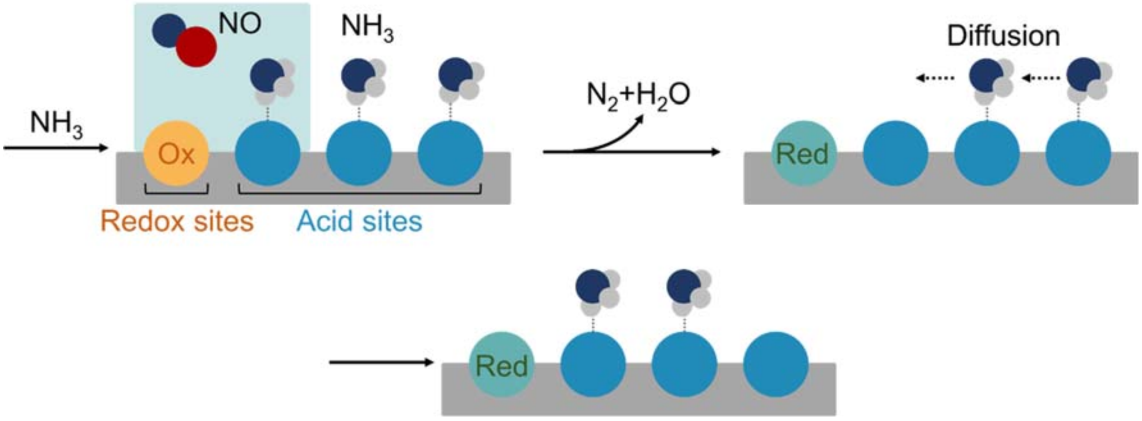

Supplementary Figure 14. *Operando* IR measurement under NO exposure. Schematic illustration of *Operando* IR measurement during NH_3 adsorption followed by NO exposure.

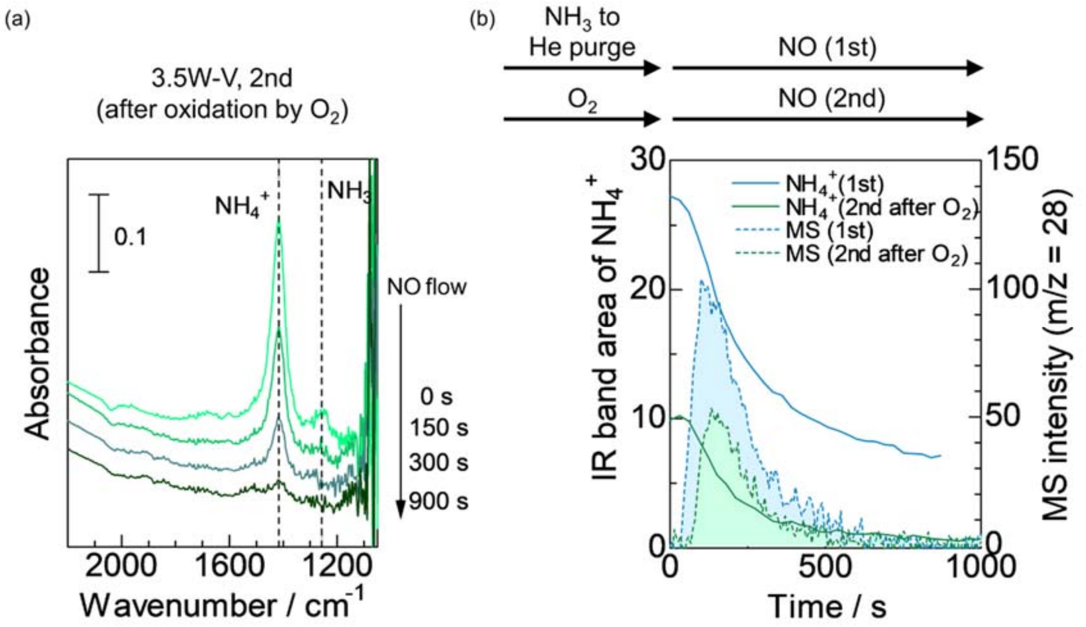

Supplementary Figure 15. ***Operando*** **IR measurement for 3.5W-V (NH_3 → 1st NO → O_2 → 2nd NO).** (a) *Operando* IR spectra of ad-species on 3.5W-V (bulk W-substituted vanadium oxide) during second NO exposure at 200ºC. The IR disc was exposed to 0.1% NH_3/He flow in the presence of 2% water vapor (30 min) and purged with He (20 min), followed by exposure to 500 ppm NO (He balance) flow (first NO). Then, oxygen was introduced at 200ºC (30 min) and 500 ppm NO (He balance) flowed again (second NO). (b) IR peak areas of ammonia species adsorbed on Brønsted acid sites (NH_4^+) and MS intensity of N_2 versus time of first (blue) and second (green) NO flowing. The curves and spectra for the first NO flowing correspond to those in figure 10c in the main text.

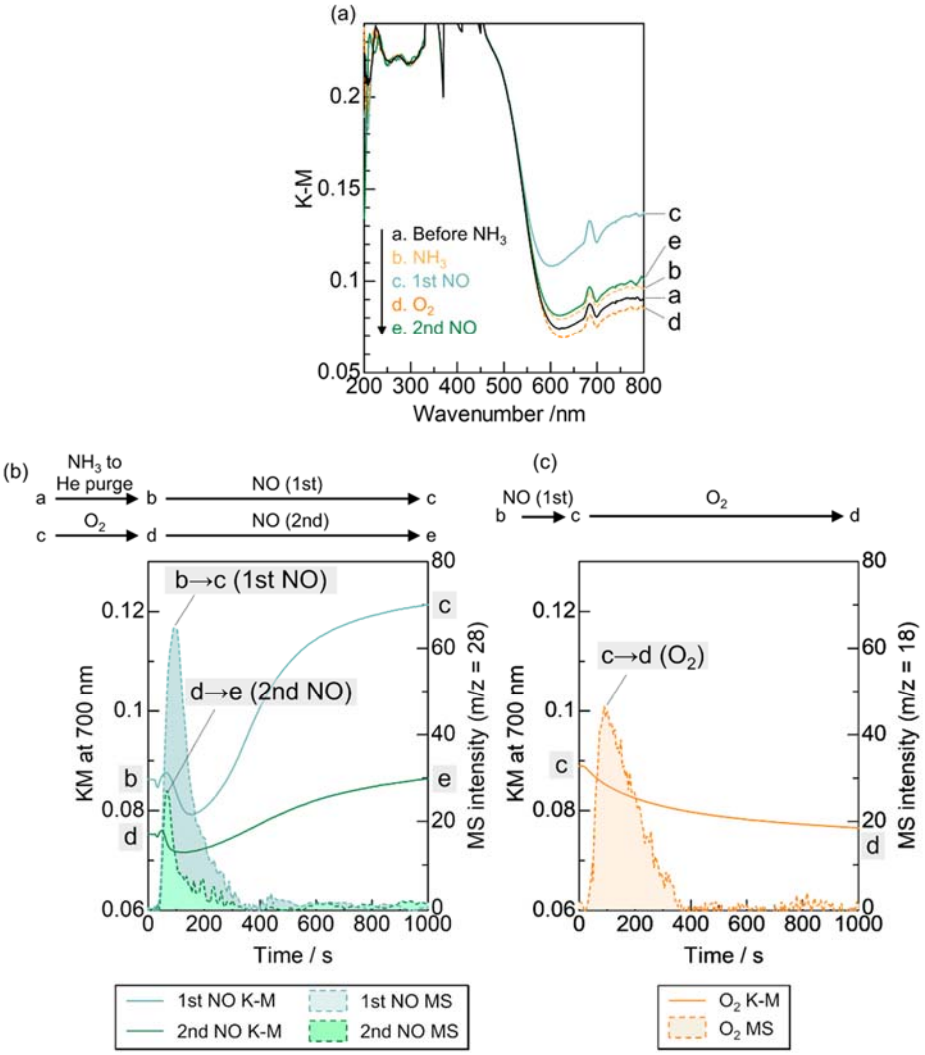

Supplementary Figure 16. ***Operando*** **UV-Vis measurements for 3.5W-V (NH_3 → 1st NO → O_2 → 2nd NO).** (a) *Operando* UV-Vis spectra of 3.5W-V after NH_3 adsorption → first NO exposure (500 ppm NO) → oxygen exposure (8% O_2) → second NO exposure (500 ppm NO) at 200ºC. (b) Change in the Kubelka-Munk unit at λ=700nm and MS intensity of N_2 (m/z = 28) as function of time for 3.5W-V during second and first NO (500ppm) flowing at 200ºC. (c) Change in the Kubelka-Munk unit at λ=700nm and MS intensity of H_2O (m/z = 18) as function of time for 3.5W-V during oxygen (8%) flowing at 200ºC. The sample was exposed to 0.1% NH_3/He flow in the presence of 2% water vapor (3 min) and purged with He (1 min), followed by exposure to 500 ppm NO (He balance) flow (1000 s, first NO). Then, the sample was oxidized at 200ºC (30 min) and 500 ppm NO (He balance) flowed again (1000s, second NO). The characters a-e correspond to the points at which the UV-Vis spectra were observed.

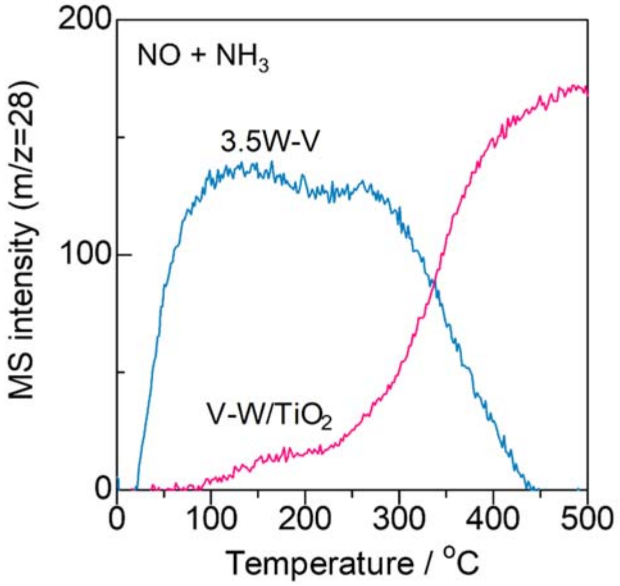

Supplementary Figure 17. Reducibility of bulk and supported W-substituted vanadium oxide catalysts. Temperature-programmed reaction spectra (TPR) of 3.5W-V (bulk W-substituted vanadium oxide) and V-W/TiO_2 (commercial supported catalyst) under NO (500 ppm) + NH_3 (500 ppm) flow. Conditions: amount of the catalyst, 40 mg; programming rate, 20ºC / min. A mass spectrometer (BELMass, MicrotracBEL Corp.) was used for analysis of N_2 gas.
